# 3D MXene‐Based Flexible Network for High‐Performance Pressure Sensor with a Wide Temperature Range

**DOI:** 10.1002/advs.202205303

**Published:** 2022-12-25

**Authors:** Yimei Xie, Yongfa Cheng, Yanan Ma, Jian Wang, Junjie Zou, Han Wu, Yang Yue, Baowen Li, Yihua Gao, Xin Zhang, Ce‐Wen Nan

**Affiliations:** ^1^ State Key Laboratory of Advanced Technology for Materials Synthesis and Processing Center of Smart Materials and Devices & International School of Materials Science and Engineering Wuhan University of Technology Wuhan 430070 P. R. China; ^2^ Wuhan National Laboratory for Optoelectronics Huazhong University of Science and Technology Wuhan 430074 P. R. China; ^3^ Hubei Key Laboratory of Energy Storage and Power Battery School of Mathematics Physics and Optoelectronic Engineering Hubei University of Automotive Technology Shiyan 442002 P. R. China; ^4^ Information Materials and Intelligent Sensing Laboratory of Anhui Province Key Laboratory of Structure and Functional Regulation of Hybrid Materials of Ministry of Education Institutes of Physical Science and Information Technology Anhui University Hefei 230601 P. R. China; ^5^ State Key Lab of New Ceramics and Fine Processing School of Materials Science and Engineering Tsinghua University Beijing 100084 P. R. China

**Keywords:** MXene, high sensitivity, piezoresistive sensor, polyetherimide, wide temperature

## Abstract

With the increasing popularity of smart wearable devices, flexible pressure sensors are highly desired in various complex application scenarios. A great challenge for existing flexible pressure sensors is to maintain high sensitivity over a wide temperature range, which is critical for their applications in harsh environments. Herein, a flexible piezoresistive sensor made of polyetherimide (PEI) fibrous network evenly covered with MXene nanosheets is reported to construct conductive pathways, showing ultrahigh sensitivity over a wide temperature range from −5 °C (sensitivity of 80 kPa^−1^) to 150 °C (20 kPa^−1^), low detection limit of 9 Pa, fast response time of 163 ms, outstanding durability over 10 000 cycles at room temperature, 2000 cycles at 100 °C and 500 cycles at −5 °C. The pressure sensor can monitor various human activities in real‐time, apply to human–machine interaction, and measure pressure distribution. It also can sensitively respond to external mechanical stimuli at 150 °C and extremely low temperature (in liquid nitrogen). Moreover, the fibrous network exhibits an excellent Joule heating performance, which can reach 78 °C at an applied voltage of 12 V. Thus, the piezoresistive sensor has considerable potential for wearable garments and personal heating applications in harsh temperature conditions.

## Graphical abstract

A highly‐sensitive piezoresistive sensor based on 3D MXene/polyetherimide network is proposed. The sensor exhibits ultrahigh sensitivity in a wide temperature range from 80 kPa^−1^ at −5 °C to 20 kPa^−1^ at 150 ^°^C, low detection limit of 9 Pa, outstanding durability over 10 000 cycles at room temperature, 2000 cycles at 100 ^°^C and 500 cycles at −5 ^°^C.

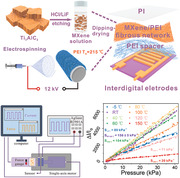

## 1 Introduction

With the flourishing development of wearable devices for applicability in human motion detection, health monitoring and artificial intelligence interaction, pressure sensors with excellent flexibility, and mechanical properties are attracting extensive attention.^[^ ^1^, ^2^, ^3^ ^]^ In particular, the wearable pressure sensors suitable for harsh conditions with high/low temperatures are eagerly pursued to meet the urgent demand for polar exploration, disaster relief, human space programs, etc. For example, to detect the real‐time health signals of astronauts on the moon, the attached flexible pressure sensor not only requires excellent sensitivity and rapid response to external stimuli, but also must be suitable to work under a wide temperature zone of extreme heat (150 °C) and cold (−150 °C) conditions.^[^ ^4^ ^]^

The current pressure sensors are mainly based on four sensing mechanisms including piezoelectric,^[^ ^5^ ^]^ capacitive,^[^ ^6^ ^]^ triboelectric,^[^ ^7^ ^]^ and piezoresistive^[^ ^8^, ^9^ ^]^ effects. Among them, piezoresistive pressure sensors, which transform the sensed mechanical pressure into electrical signals output according to a specific rule, have been widely studied and used due to their low‐cost preparation process, relatively simple readout system and good sensitivity. Traditional silicon or ceramic‐based piezoresistive sensors can operate at high temperatures,^[^ ^10^, ^11^, ^12^ ^]^ but fail to meet the requirements of flexibility, and the detection of subzero temperature is also a problem worthy of further study. The flexible piezoresistive sensors usually consist of conductive active materials and polymer substrates to acquire tension or compression elasticity. However, current substrate polymers, such as polydimethylsiloxane (PDMS),^[^ ^1^, ^13^ ^]^ polyurethane (PU),^[^ ^14^, ^15^, ^16^ ^]^ and other elastic polymers,^[^ ^17^, ^18^, ^19^ ^]^ cannot withstand high‐temperature conditions to maintain structural stability. Simultaneously achieving high sensitivity and wide temperature stability in flexible sensors remains a great challenge.

In this work, we report a high‐performance flexible piezoresistive sensor based on 3D MXene/PEI network applicable for a wide temperature range (from liquid nitrogen temperature to 150 °C). PEI is an amorphous thermoplastic polymer with high‐temperature resistance, excellent tensile strength, and superior dimensional stability, which has already displayed great potential applications in the aerospace field.^[^ ^20^, ^21^ ^]^ In addition, the PEI containing ether bonds exhibits great flexibility and processability, which can construct different micro‐ and macrostructures through various physical processes (e.g., solution casting,^[^ ^22^, ^23^ ^]^ extrusion molding,^[^ ^24^ ^]^ and electrostatic spinning method^[^ ^25^, ^26^ ^]^). On the other hand, the emerging MXenes nanosheets with metallic conductivity, excellent mechanical strength, and well hydrophilicity,^[^ ^27^, ^28^, ^29^, ^30^ ^]^ can withstand a certain low temperature (below −200 °C)^[^ ^31^ ^]^ and high temperature (over 400 °C),^[^ ^32^ ^]^ which is suitable for construction of wearable pressure sensor with PEI fibrous network applied in the harsh condition.

Relying on a layer‐by‐layer electrospinning process of PEI, 3D MXene/PEI network was fabricated to improve sensing performance in the wide temperature range. The MXene/PEI inter‐connected 3D fibrous network like a spider web was prepared, where the MXene nanosheets were coated on the surface of each PEI nanowire. Such a 3D MXene/PEI network is expected to greatly benefit large deformation under external force and improve the sensing performance of piezoresistive sensors under high‐temperature. In addition, an ultrathin PEI layer was assembled between the MXene/PEI network and flexible electrodes as a spacer, which can further enhance the conductive paths under external deformation. The obtained flexible piezoresistive sensor exhibits outstanding sensitivity over a wide temperature range (from liquid nitrogen to 150 °C), short response/recovery time, low detection limit, and excellent durability under high temperatures, which shows potential applications in body activity, health monitoring, pressure distribution arrays, and human–machine interaction. In addition, the 3D MXene/PEI fibrous network has a high Joule heating capability, reaching 78 °C at 12 V imposed voltage, which is applicable for personal heating.

## 2 Results and Discussion

### 2.1 Preparation and Characterization of MXene/PEI Piezoresistive Sensor

**Figure 1:**
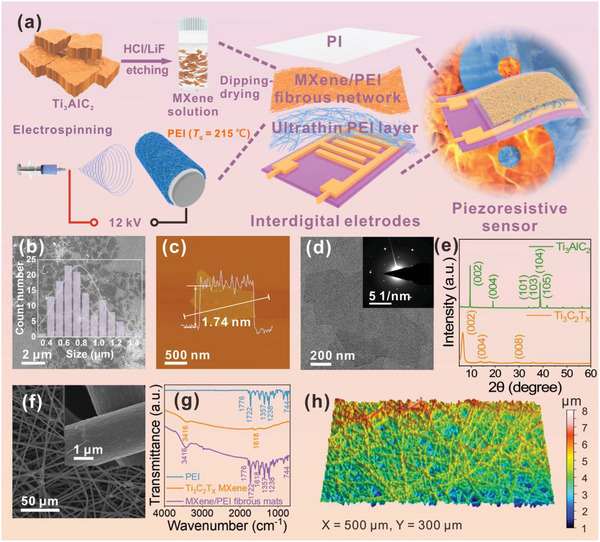
a) Schematic of the preparation process of MPP piezoresistive sensor. b) SEM image and size distribution of Ti_3_C_2_T*_x_* MXene nanosheets. c) AFM image of MXene nanosheet. The height profile indicates that the thickness of nanosheet is ≈1.74 nm. d) TEM image of MXene nanosheet, the inset shows its corresponding selected area electron diffraction pattern. e) XRD patterns of Ti_3_AlC_2_ MAX and Ti_3_C_2_T*_x_* MXene nanosheets. f) SEM image of MXene/PEI fibrous network g) Fourier transform infrared (FTIR) spectra of the PEI fibrous network, Ti_3_C_2_T*_x_* MXene, and MXene/PEI fibrous network. h) 3D structure diagram of MXene/PEI fibrous network.

**Figure** **1**a shows the detailed fabrication process of the piezoresistive sensors consisting of MXene/PEI fibrous network and ultrathin PEI layer (MPP) (see detail in the Experimental Section). To confirm the successful fabrication of MXene/PEI fibrous network, we have performed morphologies and component characterizations about the MXene nanosheets and MXene/PEI fibrous network as shown in Figure 1b–h. It is depicted that high‐quality 2D MXene nanosheets were successfully prepared with an average lateral size of 770 nm and a thickness of 1.74 nm (Figure 1b,c; and Figure S1, Supporting Information). The obtained Ti_3_C_2_T*_x_* colloidal solution showed an obvious Tyndall effect, proving its homogeneous dispersion and stability (Figure S2, Supporting Information). Transmission electron microscopy (TEM) image and selected area electron diffraction (SAED) in the inset of Figure 1d also confirmed that the MXene nanosheet is ultrathin with the typical hexagonal crystal structure. The high‐angle annular‐dark‐field (HAADF) image and related energy‐dispersive spectroscopy (EDS) indicated the uniform distribution of C, Ti, O, and F elements on the MXene nanosheet (Figure S3, Supporting Information), which also illustrated by the X‐ray photoelectron spectroscopy (XPS) in Figure S4 (Supporting Information). The prominent peak at 2*θ* = 6.86° of X‐ray diffraction (XRD) of Ti_3_C_2_T*_x_* (Figure 1e) corresponds to the characteristic (002) crystal plane, further indicating the successful fabrication of MXene nanosheets.^[^ ^33^ ^]^

The morphology of MXene/PEI fibrous network was characterized by SEM, as shown in Figure 1f. The MXene nanosheets are uniformly and tightly coated on each fiber of PEI fibrous network. The corresponding elemental maps also revealed uniform distributions of C, Ti, O, F elements (Figure S5, Supporting Information). Besides, the component of MXene/PEI fibrous network was validated by Fourier transform infrared (FTIR) in Figure 1g. The typical peaks of C=O vibration at 1776 and 1722 cm^−1^, C—N stretching vibration at 1357 and 1238 cm^−1^ were attributable to the PEI fibrous network and the O—H peak at 3416 cm^−1^ is the characteristic peak of Ti_3_C_2_T*_x_* MXene nanosheets.^[^ ^34^, ^35^ ^]^ The microstructure of MXene/PEI fibrous network was also analyzed by 3D optical profiler, as detailed in Figure 1h. It is revealed that the MXene/PEI fibrous network was randomly distributed with microscopic protrusions, allowing for the construction of highly sensitive piezoresistive sensor.

### 2.2 Working Mechanism of the MPP Sensor

**Figure 2:**
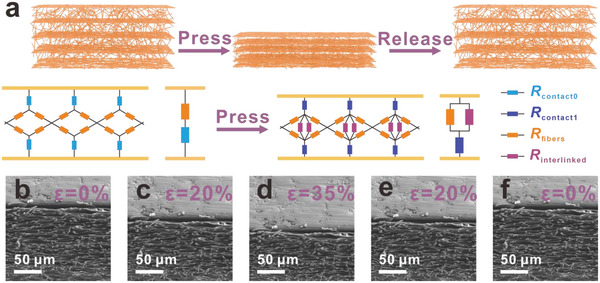
The sensing mechanism of MPP piezoresistive sensor. a) MPP sensor pressure‐sensing models that show how the network's contact area changes as a result of compressive deformation. The following model schematics is the further simplified resistance model of MPP piezoresistive sensor. The baby blue and dark blue symbols represent contact resistance between sensor and interdigital electrodes before and after compression, respectively. The orange and purple symbols represent the resistance of conductive MXene/PEI fibers, and the resistance caused by the deformation of MXene/PEI fibrous network during the external pressure, respectively. b–f) In‐situ SEM images of MXene/PEI fibrous network under different compression states.

The dynamic change of microstructural variation was measured by in situ SEM when applying and withdrawing the external force, as detailed in **Figure** **2**b–f; and Video S1 (Supporting Information). It is observed that PEI fibrous with multilayer porous network supports the robust 3D scaffold for deformation and thus displays excellent mechanical properties. As shown in Figure 2a, by combing the in situ SEM experiment results, a schematic model of the MXene/PEI sensor is proposed to reveal the relevant work mechanism.

In the initial state without applied pressure, the fibrous network has a rich porous structure (Figure 2b; and Figure S6, Supporting Information), and there is only a little contact area between the conductive fibers. The overall equivalent circuit initial resistance (*R*_0_) can be described as follows

(1) $$R_{0}=R_{\mathrm{contact}0}+R_{\mathrm{fibers}}$$

where *R*_contact0_ is the contact resistance between interdigital electrodes and conductive fibers, *R*_fibers_ is the resistance of conductive fibers. As shown in Figure S7 (Supporting Information), finite element analysis (FEA) was used to simulate the deformation of the pressure sensor under external pressure. As pressed against the sensor, the pressure is well transmitted and distributed to fibers of the PEI fibrous network, leading to the close contact of the fibers.

As the external pressure increases, a high stress distribution on the fibers leads to a large deformation and a continuous increase in conductive pathways between the fibers, resulting in a further increase of current change. The resistance of new conductive pathways is defined as *R*_interlinked_, which is linked with the initial resistance of conductive pathways in parallel. In addition, the contact area between the electrodes and conductive fibers increases with the application of external pressure, leading to the change of the contact resistance. *R*_contact1_ is defined as contact resistance of the electrodes and conductive fibers during compression. The overall equivalent circuit resistance (*R*) can be described as follows

(2) $$R=R_{\mathrm{contact}1}+\frac{R_{\mathrm{fibers}}R_{\mathrm{interlinked}}}{R_{\mathrm{fibers}}+R_{\mathrm{interlinked}}}$$

where *R*_contact1_ is defined as contact resistance of the electrodes and conductive fibers during compression.

The application of an external force will lead to more contact areas between PEI fibrous network and interdigital electrodes, and new conductive pathways in MXene/PEI fibrous network generate, which determine the resistance change of the sensor. When the external pressure was withdrawn quickly, the MXene/PEI fibrous network almost return to the original state immediately, leading to a sharp decrease of the conductive pathways and the contact areas. The sensitivity of MPP sensor can be expressed as

(3) $$S=\frac{\Delta I/I_{o}}{\Delta p}=-\frac{\Delta R/R}{\Delta p}=-\frac{\left(\frac{R_{\mathrm{fibers}}R_{\mathrm{interlinked}}}{R_{\mathrm{fibers}}+R_{\mathrm{interlinked}}}-R_{\mathrm{fibers}}+\Delta R_{\mathrm{contact}}\right)/(R_{\mathrm{contact}1}+\frac{R_{\mathrm{fibers}}R_{\mathrm{interlinked}}}{R_{\mathrm{fibers}}+R_{\mathrm{interlinked}}})}{\Delta p}$$

where Δ*R*_contact_ = *R*_contact1_ − *R*_contact0_, indicating he change of contact resistance, Δ*I* stands for the change in current during applying and withdrawing the external force, *I*_o_ is the original current without loading pressure, Δ*P* represents the change of the applied pressure. The unique and reversible microstructure of the MXene/PEI fibrous network endows the sensor with the excellent sensing performances described below.

### 2.3 Sensing Performance of the MPP Sensor

**Figure 3:**
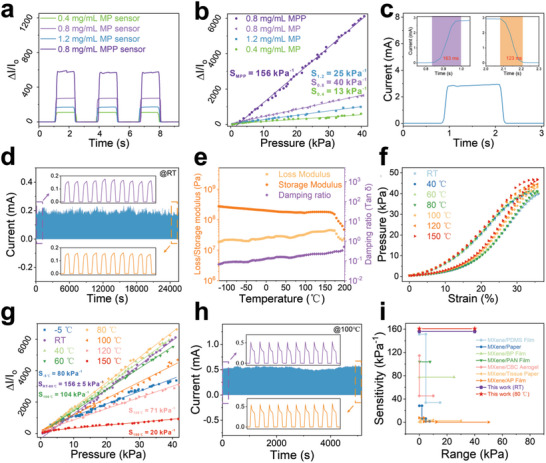
The sensing performances of MPP piezoresistive sensor. a) Current response Δ*I*/*I*_o_ versus time. b) The relative change of the current (Δ*I*/*I*_o_) as the pressure load changes for the MXene/PEI sensors with different MXene concentrations. c) The response time (163 ms) and recovery time (123 ms) of MPP sensor at compression speed of 200 mm s^−1^. d) The durability test of the sensor under 10 000 continuous loading‐unloading cycles. e) Dynamic rheological behavior of MXene/PEI fibrous network from −120 to 200 °C. f) The stress–strain curves of MXene/PEI fibrous network and g) the sensitivities of MPP sensor under different temperature conditions from RT to 150 °C. h) The cyclic piezoresistive sensing performance of the MPP sensor at 100 °C over 2000 cycles. i) Comparison of sensitivity and sensing range for different piezoresistive sensors.

To study the piezoresistive effect of the MXene/PEI fibrous network, we used high‐precision force/electrical test system to measure the current and force signals. The MXene/PEI fibrous network piezoresistive (MP) sensors have been prepared with various starting MXene concentrations of 0.4, 0.8, and 1.2 mg mL^−1^. In **Figure** **3**a, the sensor fabricated with MXene concentration of 0.8 mg mL^−1^ has the greatest on/off ratio of current under the same pressure value (5.8 kPa), indicating that it has higher sensing performance than others with MXene concentrations of 0.4 and 1.2 mg mL^−1^. The microstructure of PEI fibrous network with different MXene concentrations were visually displayed in Figure S8 (Supporting Information). And the MXene coating on the PEI fibers thickens with increasing MXene concentration. In principle, as the concentration of MXene increases, the current change of sensors steadily improves, because the increase of MXene coating on PEI fibrous network can effectively create more conductive pathways and significantly improve the capability of detecting tiny pressure.^[^ ^17^ ^]^ However, when the concentration of MXene solution exceeds a certain range, the MXene coating on PEI fibrous network is too thick, resulting in the relatively high initial current of the sensor. Under the external stimulation, the conduction paths approach saturation, in turn leading to relatively small current changes. And compared with the 0.8 mg mL^−1^ MP sensor, the MXene/PEI fibrous network and ultrathin PEI layer (MPP) device exhibits a higher current switch ratio, in Figure 3a. It indicates that the inserted PEI layer (Figure S9, Supporting Information) between the sensor is conducive to improving the sensing performance.

Figure 3b shows the relative change of the current (Δ*I*/*I*_o_) as the applied pressure changes for the MXene/PEI sensors with different MXene concentrations. The sensitivity of the MP sensor with 0.8 mg mL^−1^ MXene is higher than the sensors fabricated with 0.4 and 1.2 mg mL^−1^ MXene. And the sensitivity of MPP sensor is as high as 156 kPa^−1^ in the pressure region from 0 to 40 kPa (Figure 3b), which is higher than that of the MP sensor over the entire test pressure range. The trend is completely consistent with the on/off ratio of current in Figure 3a. In order to analyze and compare the initial current, we have tested the *I–V* curves for MP and MPP‐based sensors without the applied pressure in Figure S10 (Supporting Information). It is observed that the initial current of MP sensor shows a mild increase with the increasing the MXene concentration from 0.4 to 0.8 mg mL^−1^, but a dramatical increase from 0.8 to 1.2 mg mL^−1^. The MP sensor with MXene concentration of 1.2 mg mL^−1^ exhibits a remarkably higher initial current than the counterparts with 0.4 and 0.8 mg mL^−1^. After inserting the ultrathin PEI layer, the initial current of the sensor (MPP sensor) shows a significant decrease compared to the MP sensor with the same MXene concentration of 0.8 mg mL^−1^, which is consistent with the above explanation.

Figure S11 (Supporting Information) displays the *I–V* curves of MPP sensor under different pressures. The linear relationship suggests a good ohmic contact between the sensor and interdigital electrodes. And the slope of *I–V* curves increases with the increase of the pressure, indicating that the sensor can distinguish different levels of pressure, which is also illustrated in Figure S12 (Supporting Information). Response/recovery time is also an important parameter to evaluate the sensing performance. The MPP sensor shows rapid response time of 163 ms and recovery time of 123 ms in Figure 3c, allowing it to quickly respond to the external stimulus. *I–t* curves of the sensor responded consistently at different frequencies (Figure S13a, Supporting Information) and speed (Figure S13b, Supporting Information) when applied with the same force. Meanwhile, the sensor represented exceptional stability and durability during 10 000 loading and unloading cycles under pressure of 0.8 kPa (Figure 3d), and there is little degradation in electronic signal after 6000 loading–unloading cycles at high pressure of 25.82 kPa in Figure S14 (Supporting Information). The SEM characterizations reveal that the morphology and structure of sensing element have no change during the cycle tests (Figure S15, Supporting Information). Meanwhile, the current has no substantial change, demonstrating the superior stability of the sensor. The device can still maintain almost same current response under the same pressure after 12 days, which proves again its excellent stability (Figure S16, Supporting Information). And we also repeatedly tested six MPP sensors under different pressure range, as shown in Figure S17 (Supporting Information). It is observed the current response of each MXene/PEI fibrous‐based sensor is basically the same under the same applied pressure, which proves the good consistency of the devices.

To further gain insights into the applicability of the MXene/PEI sensor in harsh conditions, we carried out a series of tests in a wide temperature range, as shown in Figure 3e–h. The viscoelastic characteristics of the MXene/PEI fibrous network are essentially constant over a temperature range of −120 to 200 °C (Figure 3e), demonstrating its outstanding structural stability across a wide temperature range. Additionally, the MXene/PEI fibrous network exhibits exceptional elasticity owing to its low damping ratio, which shows that it loses minimal energy during dynamic deformation. The stress–strain curves at different temperatures were subsequently performed to evaluate the high‐temperature tolerance in Figure 3f. The stress–strain curves from RT to 80 °C almost overlap each other, showing temperature‐independent mechanical properties. The excellent temperature stability of PEI network indicates promising adaptability of the flexible sensor under harsh temperature environments. As shown in Figure 3g, from RT to 80 °C, the output current signal of the pressure sensor keeps the same trend (Figure S18, Supporting Information), and the sensitivity remains almost the same at 156 ± 5 kPa^−1^. From 100 to 150 °C, the sensitivity monotonically decreases from 104 kPa^−1^ at 100 °C, 71 kPa^−1^ at 120 °C to 20 kPa^−1^ at 150 °C, which still has excellent sensing performance. And the sensitivity of the sensor remains a high level of 80 kPa^−1^ at −5 °C. In addition, the response time and recovery time of the MPP pressure sensor are measured in different temperature in Figure S19 (Supporting Information). In the temperature range of −5 to 150 °C, the response time of the device is 123–176 ms, and the recovery time is 93–123 ms. This data difference is mainly attributed to the experimental error and the different deformation capabilities of the sensing element under cold and hot environments. Subsequently, long‐term cyclic compression tests in hot and cold conditions were conducted to verify the fatigue resistance of the piezoresistive sensor in different temperature. There is little performance degradation of the sensor after 2000 loading and unloading cycles, which exhibits its excellent performance under high temperature of 100 °C in Figure 3h. The MPP sensor also displays good durability under low temperature (−5 °C) in Figure S20 (Supporting Information). After high‐temperature test, the MXene/PEI fibrous network has only slight oxidation (Figure S21, Supporting Information), but no morphology and structure change (Figure S22, Supporting Information). As indicated in Figure 3i; and Table S1 (Supporting Information), the advantages of high sensitivity within a wide linear range and outstanding temperature tolerant performance are superior to other reported MXene piezoresistive sensors,^[^ ^1^, ^36^, ^37^, ^38^, ^39^, ^40^, ^41^ ^]^ which are expected to greatly widen the applications of the flexible pressure sensor.

### 2.4 Applications of the MPP Sensor

**Figure 4:**
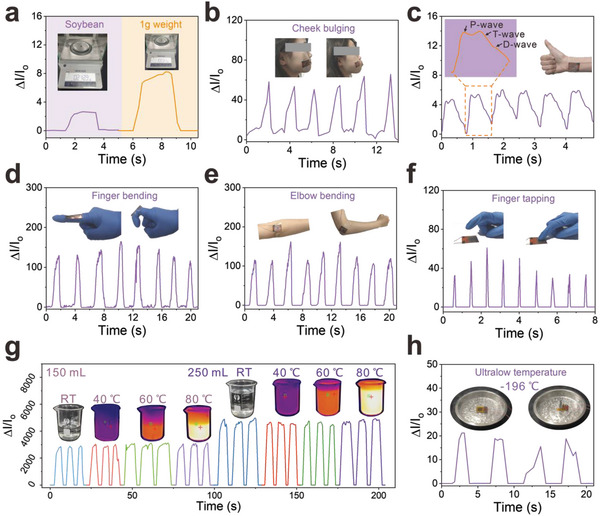
Application of the MPP piezoresistive sensor. The response curves of a) tiny object pressure provided by a soybean (9 Pa) and 1 g weight (44 Pa), b) cheek bulging, c) wrist pulse (the enlarged illustration is a single pulse signal that contains characteristic peaks called P‐wave, T‐wave, and D‐wave), d) finger bending, e) elbow swing, f) finger tapping, and g) water glasses holding at different temperatures from RT to 80 °C. h) Sensing performance of MPP piezoresistive sensor under ultralow temperature (liquid nitrogen).

Taking the advantages of high sensitivity and fast response, the MXene/PEI piezoresistance sensor is applicable to monitor subtle pressures and different human activities in real‐time. The detection limit of a piezoresistive sensor is one of the most critical performance indicators. As illustrated in **Figure** **4**a, the sensor can detect tiny pressures given by a soybean (9 Pa) and a weight of 1 g (44 Pa). Moreover, the flexible sensor can be conformally attached to human muscles. As displayed in Figure 4b, the minimal muscle movement of cheek bulging can be recorded. Wrist pulse, as a faint vital sign, is relatively challenging for accurate detection. In Figure 4c, the sensor was directly fastened to the wrist skin of an adult male to real‐time monitor the pulse rate and waveform. Noticeably, the pulse rate is recorded at 73 beats per minute. And it is simple to discern the three typical peaks, P, T, and D, which stand for percussive, tidal, and diastolic waves, respectively. So the sensor is capable of recording the wrist pulse and clearly distinguishing the waveforms, which have great promise for biomedical monitoring and real‐time clinical diagnosis.

Due to the flexibility, the MPP piezoresistance sensor can also be tightly attached to human joints. As shown in Figure 4d, the sensor can detect the bending and straightening movements of an index finger in real‐time. As illustrated in Figure 4e and Figure S23 (Supporting Information), the sensor also shows high sensitivity to monitor the flexion‐extension movement cycles of human joints, such as wrist, elbow, ankle, and knee, respectively. In Figure 4f, the sensor also presents rapid response of high‐frequency finger tapping.The shape and intensity of currents can distinguish different magnitudes of forces.

Due to its remarkable temperature stability, the MPP piezoresistance sensor shows almost the same electrical signal when the experimental volunteer holds 150 mL water cups at RT, 40, 60, and 80 °C (Figure 4g). Figure S24 (Supporting Information) displayed how the sensors were mounted and how the experiments were tested. When the volume of water is increased to 250 mL, the signal response to different temperatures is enhanced synchronously. Furthermore, even in extremely cold temperature like liquid nitrogen, the sensor exhibits stable sensing capability (Figure 4h). The sensor can also detect the holding of cups with different volumes of liquid nitrogen, as shown in Figure S25 (Supporting Information). The current signal responds well to the change of weight of the cup at an ultralow temperature of around the liquid nitrogen temperature. The broad working temperature range shows the application potential of our sensor in the aerospace field.

**Figure 5:**
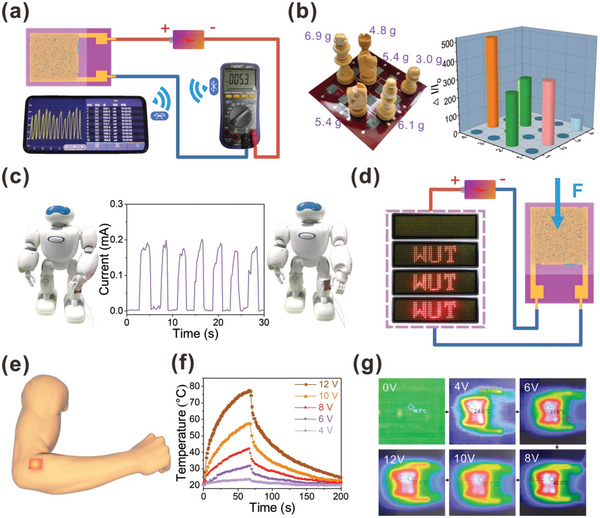
a) The MPP piezoresistive sensor is linked to a circuit included a Bluetooth module, which transfers wirelessly signal of finger tapping to the mobile phone. b) Chess pieces are placed on the 4×4 MPP piezoresistive sensor pixel arrays, and the corresponding pressure distributions were detected. c) The real‐time sensing for monitoring robot movements by the MPP piezoresistive sensor. d) The brightness response of LED varied with different applied pressure on the MPP piezoresistive sensor. e) Schematic diagram of the MXene/PEI fibrous network attached to skin surface for Joule heating. f) Temperature profiles of the MXene/PEI fibrous network with concentration of 0.8 mg mL^−1^ under different operation voltages. g) IR thermal images of the MXene/PEI fibrous network with concentration of 0.8 mg mL^−1^ at stepwise voltage rise from 0 to 12 V.

The Internet of Things (LoT) is the core of information age, which is formed by the combination of various sensors. To further investigate the application in the field of LoT, the MPP piezoresistance sensor is connected to a Bluetooth system. As shown in **Figure** **5**a, the system can transform the sensed pressure signal into wireless electromagnetic wave signals. Then the changes in current can be monitored and recorded on the phone. Moreover, the high‐resolution flexible sensor array receives a lot of interest, which is highly required for evaluating spatially pressure in wearable devices or human–machine interface applications. We fabricated a 4×4 pixel array of MPP piezoresistive sensor and six chess pieces were placed in different positions. The schematic model of 4×4 sensing array is depicted in Figure S26 (Supporting Information). As shown in Figure 5b, by monitoring the strength of current at each pixel point, the sensor array can quantify pressure distribution to determine the particular location of chess pieces. In addition, the highly flexible sensor is mounted to a robot's leg, as schematically shown in Figure 5c. The sensor generates response signal when the robot walks with its arm swinging. And the sensor also can monitor the load of heavy objects for early warning. In detail, the sensor was connected with LED in series, getting obvious visualization from different pressure responses. The brightness of the light increases with increasing pressure in Figure 5d, due to the gradually increased current of the MPP piezoresistive sensor. Therefore, the MPP piezoresistive sensor has a broad application prospect in LoT and artificial intelligence field.

In addition to the above fascinating functions, the good electrical conductivity of MXene and high thermal stability also endow the MXene/PEI fibrous network with excellent Joule heating performance, which can be utilized as wearable heaters for thermotherapy, as shown in Figure 5e. Following the equation $Q=\frac{U^{2}t}{R}$ (*Q* is generated heat in Joule, *U* is the input direct‐current (DC) voltage, *R* is resistance, *t* is time), the steady‐state temperature increase with increasing DC voltages. Figure 5f displays the temperature profiles of a heater made of MXene/PEI fibers under DC voltages of 4, 6, 8, 10, and 12 V as measured by an infrared thermal imager. There is no observable temperature rise at 4 V, and at voltage of 6 V, the temperature increases to 32 °C. A voltage of 8 V produces a high steady‐state temperature around 42 °C, and further increases respectively to 58 and 78 °C at 10 and 12 V. The infrared (IR) thermal picture presented in Figure 5g also shows the steady‐state temperature of voltage from 0 to 12 V. As illustrated in Figure S27a (Supporting Information), the temperature is roughly proportional to the square of the applied voltage, which follows Joule's law. In order to evaluate the heating performance stability, we respectively perform loading and unloading of voltage cycles on the fibrous network under a voltage of 6, 8, 10 V. As shown in Figure S27b (Supporting Information), the little temperature fluctuations sufficiently reveal the outstanding cycling stability of MXene/PEI fibrous network. Consequently, the great Joule heating response combined with remarkable cycling stability expands the multifunctional textile material.

## 3 Conclusion

In this work, we have prepared a 3D MXene/Polyetherimide network‐based highly sensitive piezoresistive sensor. The sensor exhibits ultrahigh sensitivity in a wide temperature range (from 80 kPa^−1^ at −5 °C, 156 kPa^−1^ at RT, and 20 kPa^−1^ at 150 °C), a low detection limit of 9 Pa, a fast response time of 163 ms, and long‐term durability over 10 000 cycles for RT, 2000 cycles at 100 °C and 500 cycles at −5 °C. The pressure sensor can track different human activities in real‐time, detect pressure distribution, and be used in human–machine interactions. It can also respond sensitively to external mechanical stimuli at both high (150 °C) and low (liquid nitrogen) temperatures. Furthermore, the fibrous network has a good Joule heating capability, reaching 78 °C at a voltage of 12 V. Accordingly, the flexible MXene/PEI fibrous network has prospective applications in the fields of flexible wearable electronics devices and personal heating systems in harsh conditions.

## 4 Experimental Section

### Synthesis of MXene (Ti_3_C_2_T*_x_*) Nanosheets

Ti_3_C_2_T*_x_* MXene nanosheets were obtained using a mild route to selectively etching Ti_3_AlC_2_ MAX according to the previous reports.^[^ ^36^, ^42^ ^]^

### Fabrication of the Interdigital Electrodes

An inkjet printer was first used to fabricate a predesigned interdigital pattern (gray) on a clean polyimide (PI) film (purple). Then, after the copper evaporation deposition, the copper metal (yellow) completely covers the PI film. The ink prepattern (gray) acts as a mask plate, which has weak binding force with PI film. The copper attached to the ink pattern is easy to fall off through water washing, while the copper attached to the area without ink pattern can be retained on the PI film, thus the interdigital copper electrodes can be fabricated. The specific parameters of the interdigital electrodes are shown in Figure S28 (Supporting Information).

### Fabrication of PEI Fibrous Network and Ultrathin PEI Layer

4 g PEI pellets were dissolved into 10 mL 1‐methyl‐2‐pyrrolidinone (NMP, Aladdin, 99.9%) for 48 h at 40 °C. The stable and homogeneous solution was then transferred into a syringe. Then, the electrostatic spinning was used to fabricated by the electrospinning device (ET‐2535D) under electric field of 1.2 kV cm^−1^, and the syringe pump rate was set as 1 mL h^−1^. The PEI fibrous network was collected by the Al foil onto the surface of the winding roller. The PEI fibrous network and ultrathin PEI layer were, respectively, spun for 1 h and 60 s.

### Assembling of MXene/PEI Fibrous Network (MP) and MXene/PEI Fibrous Network/Ultrathin PEI Layer (MPP) Piezoresistance Sensor

The MXene/PEI fibrous network was fabricated using a simple dipping and drying method. The plasma cleaner administered the plasma treatment to the PEI fibrous network for 1 min to increase its hydrophilicity (Figure S29, Supporting Information). To optimize the sensing performance, different concentrations of Ti_3_C_2_T*_x_* suspension (0.4, 0.8, 1.2 mg mL^−1^) were used to prepare the sensing elements. In detail, a piece of MXene/PEI fibrous network with suitable size (1.5 × 1.5 cm^2^) was fixed onto the Cu‐coated interdigital electrodes with prespun ultrathin PEI layer, then it was properly encapsulated in a plastic film. The specifications of the MXene/PEI fibrous network/ultrathin PEI layer based sensor (MPP sensor) are shown in Figure S30 (Supporting Information). Copper wires were fastened with conductive adhesive tape at one end of the electrodes. As a comparison, the MXene/PEI fibrous network based sensor without the introduction of the ultrathin PEI layer (MP sensor) was also prepared with the same process.

### Material and Microstructure Characterization

The crystal structure of powders samples was detected by X‐ray diffraction (XRD, Rigaku Smartlab). The chemical elements and surface bonding of MXene/PEI fibrous network were performed by X‐ray photoelectron spectrometer (XPS, ESCALAB 250Xi). The morphology of MXene nanosheets was examined by transmission electron microscopy (TEM, Talos F200S). The microstructure of MXene nanosheets, ultrathin PEI network and MXene/PEI fibrous network were observed with scanning electron microscopy (SEM, JSM 7610F Plus). The thickness of MXene nanosheet was characterized using atomic force microscopy (AFM, Cypher ES) in a standard tapping mode. The morphology of the MXene/PEI fibrous network was characterized by 3D optical profiler (Nanovea ST400). The in situ SEM for press‐release dynamic process was tested by using Zeiss Crossbeam 550 FIB‐SEM.

### The Performances Characterization

The electrical property and sensing performance were measured by an electrical testing system mainly composed of a program‐controlled sport propulsion device, a force gauge, and an Agilent B2901A source meter. The input voltage was 1 V. The high‐temperature test was carried out with a flexible heating substrate. The low‐temperature test was carried out with a semiconductor cooling chip, which operates at the DC voltage of 12 V and controls the temperature with a digital temperature controller. A system combining metal and water was employed to improve the heat dissipation of the semiconductor cooling chip, thus the test condition with the minimum temperature of −5 °C wasobtained. The stress–strain curves were measured on a testing machine equipped with a computer‐controlled sport propulsion device and a dynamometer. Dynamic mechanical analysis was performed using a dynamic mechanical analyzer (DMA 8000) with 5 °C min^−1^ heating stepwise from −120 to 200 °C under a frequency of 1 Hz. The Joule heating performance was recorded by the variation of surface temperature captured by thermal imaging camera (Fluke).

## Conflict of Interest

The authors declare no conflict of interest.

## Supporting information

Supporting Information

Supplemental Video 1

## Data Availability

The data that support the findings of this study are available from the corresponding author upon reasonable request.
