# Supplementary material for: 3D MXene‐Based Flexible Network for High‐Performance Pressure Sensor with a Wide Temperature Range
Source: Adv Sci (Weinh). 2022 Dec 25;10(6):2205303. doi: 10.1002/advs.202205303 (PMC9951356; doi:10.1002/advs.202205303)
Supplement: Supplementary file 1 — Supporting Information [file ADVS-10-2205303-s002.pdf]

ADVANCED SCIENCE
Open Access

Supporting Information

for *Adv. Sci.*, DOI 10.1002/advs.202205303

3D MXene-Based Flexible Network for High-Performance Pressure Sensor with a Wide Temperature Range

Yimei Xie, Yongfa Cheng, Yanan Ma, Jian Wang, Junjie Zou, Han Wu, Yang Yue, Baowen Li, Yihua Gao, Xin Zhang* and Ce-Wen Nan**

Supporting Information

3D MXene-based flexible network for high performance pressure sensor with a wide temperature range

Yimei Xie, Yongfa Cheng, Yanan Ma, Jian Wang, Junjie Zou, Han Wu, Yang Yue, Baowen Li, Yihua Gao, Xin Zhan*, Ce-Wen Nan**

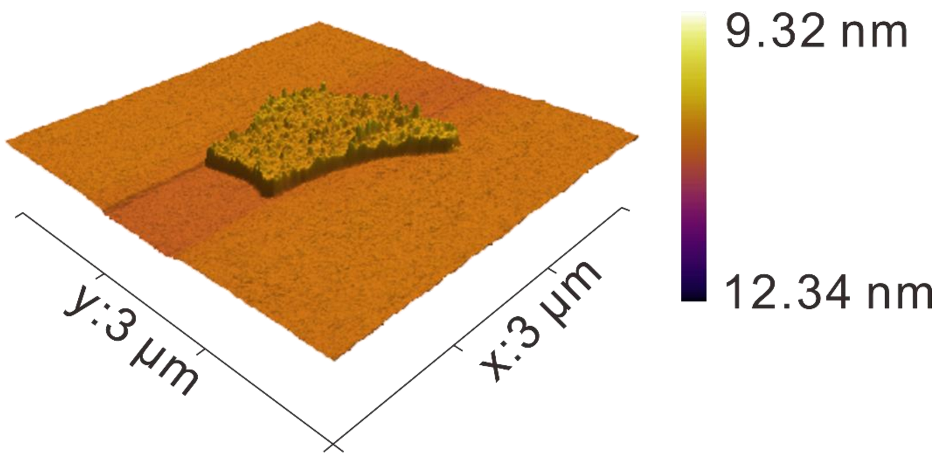

Figure S1. 3D AFM image of a specific monolayer $Ti_3C_2T_x$ MXene nanosheet.

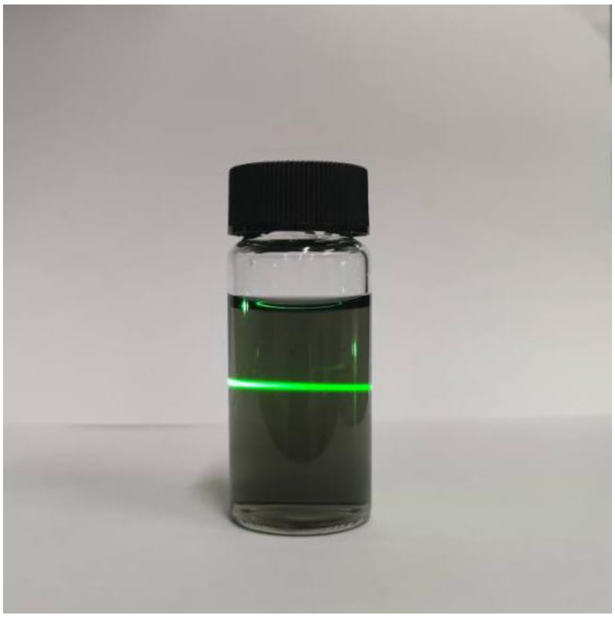

Figure S2. The Tyndall effect of $Ti_3C_2T_x$ MXene solution.

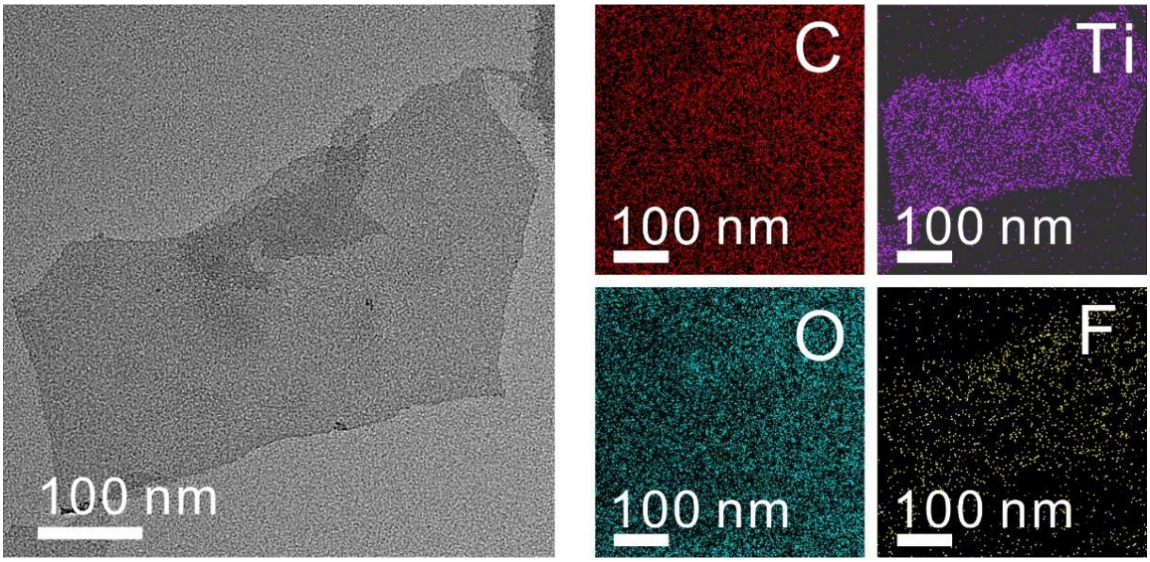

Figure S3. HAADF STEM image of a monolayer $Ti_3C_2T_x$ MXene nanosheet and the EDX elemental mapping at the same region (red: C, purple: Ti, green: O, yellow: F).

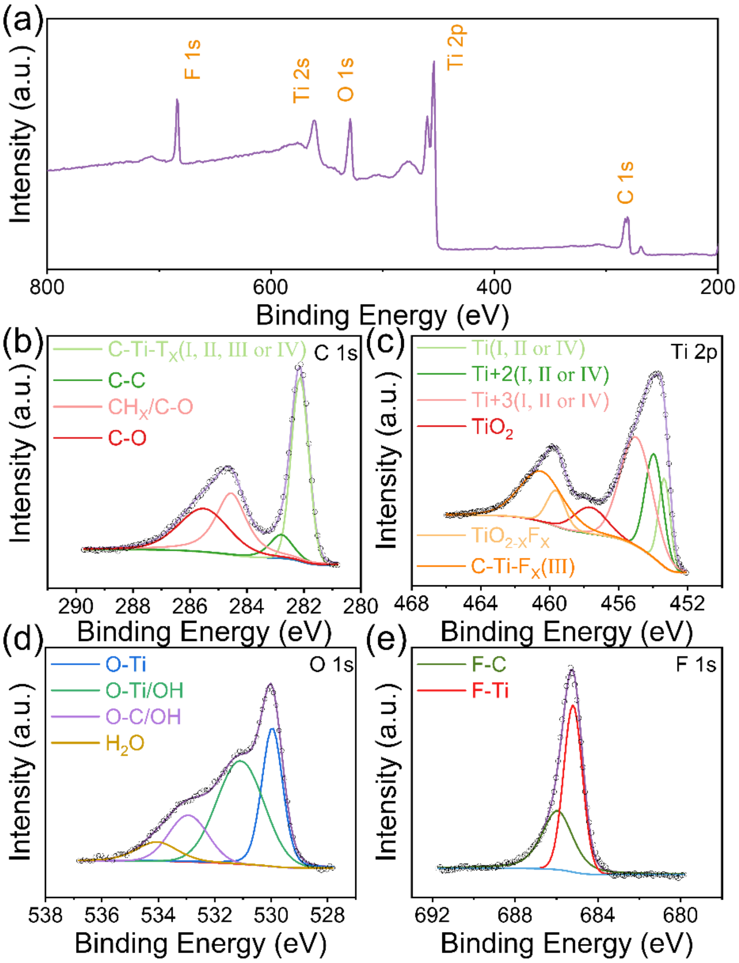

Figure S4. X-ray photoelectron spectroscopy (XPS) analysis. (a) XPS survey spectrum, and high-resolution spectra of (b) C 1s, (c) Ti 2p, (d) O 1s, and (e) F 1s of $Ti_3C_2T_x$ MXene.

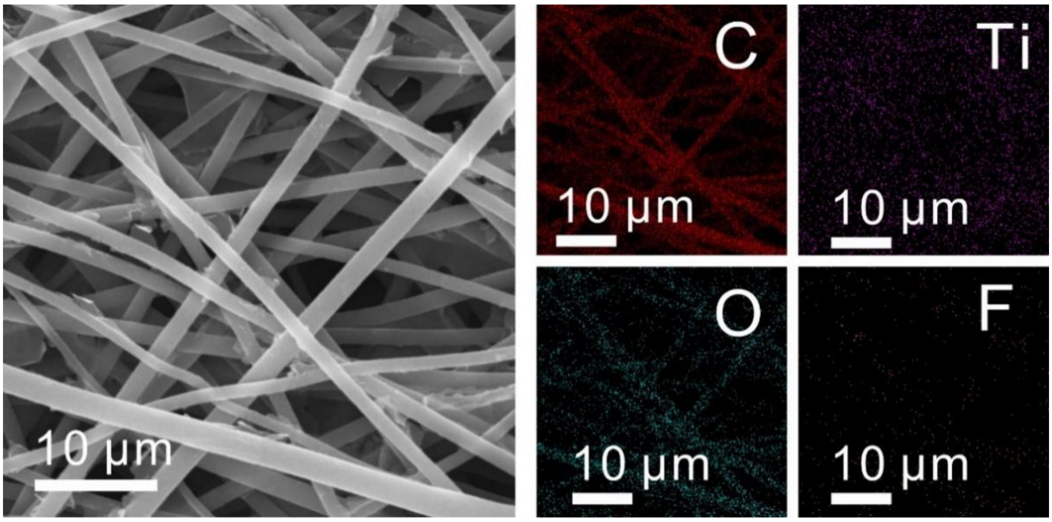

Figure S5. SEM image of MXene/PEI fibrous network and the relevant elemental mapping images (red: C, purple: Ti, green: O, yellow: F).

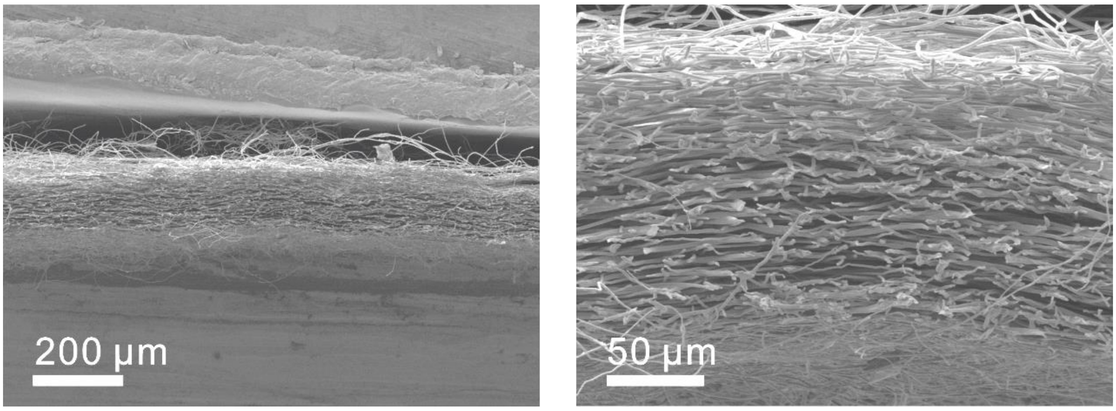

Figure S6. Cross-sectional SEM images of MXene/PEI fibrous network.

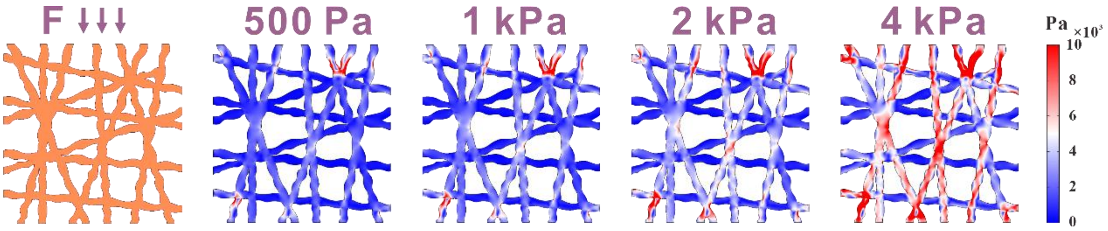

Figure S7. Simulated stress distribution of PEI fibrous network in a range of 0-4.0 kPa.

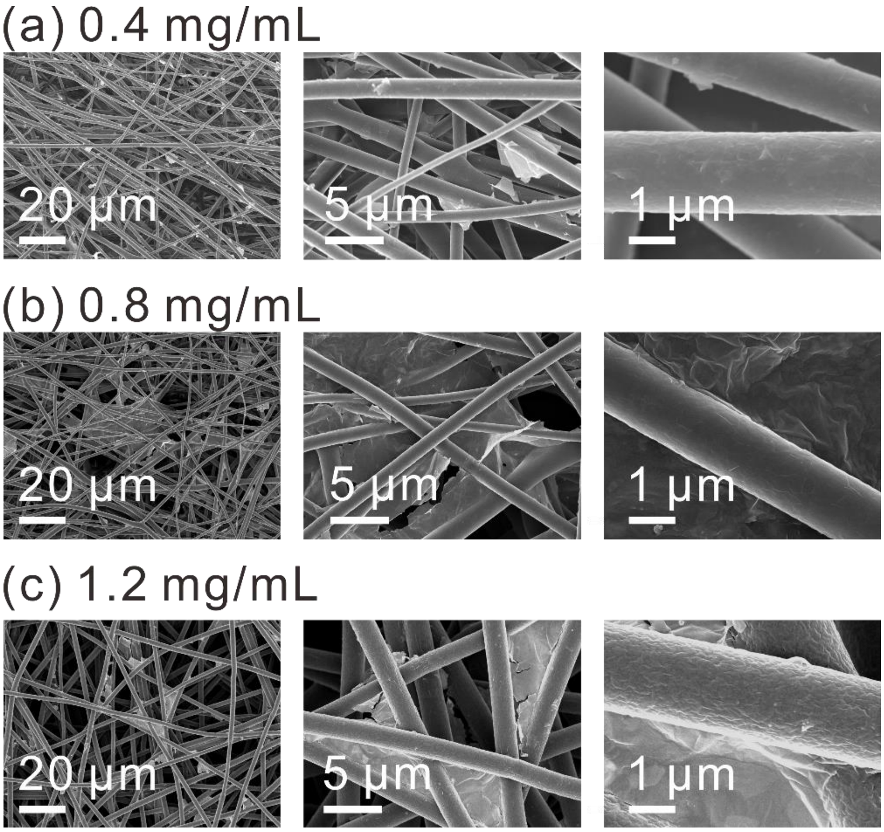

Figure S8. SEM images of the MXene/PEI fibrous network with different MXene diffraction.

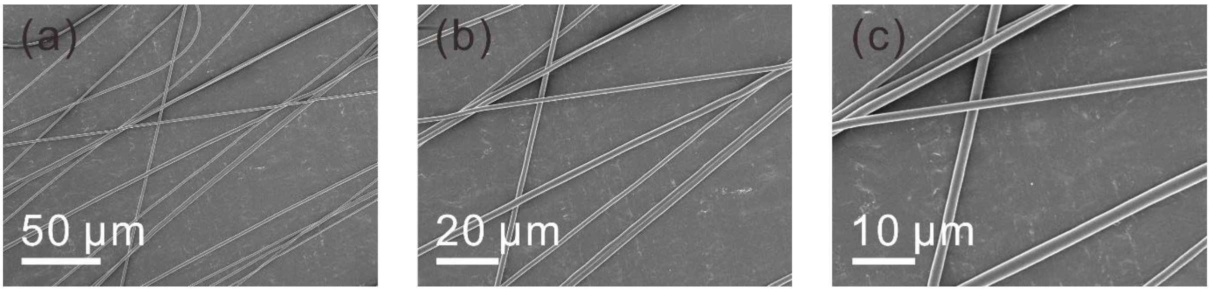

Figure S9. SEM images of ultrathin PEI layer with different magnifications.

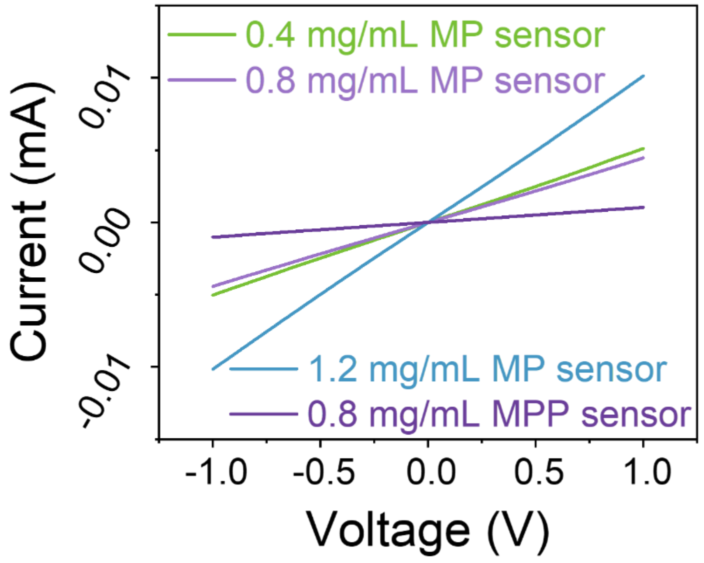

Figure S10. The I-V curves for MPP sensor and MP sensors with different concentration without applied pressure.

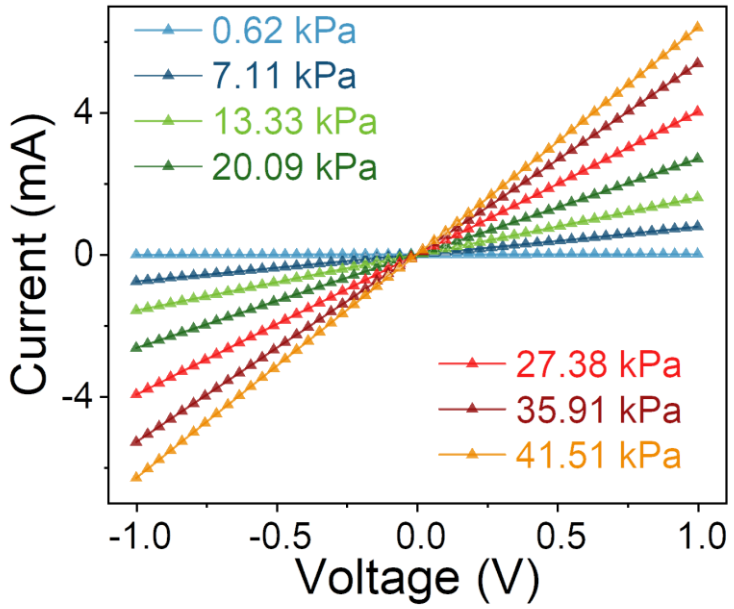

Figure S11. *I-V* curves for the sensor fabricated by 0.8 mg/mL MXene concentration below 41.51 kPa.

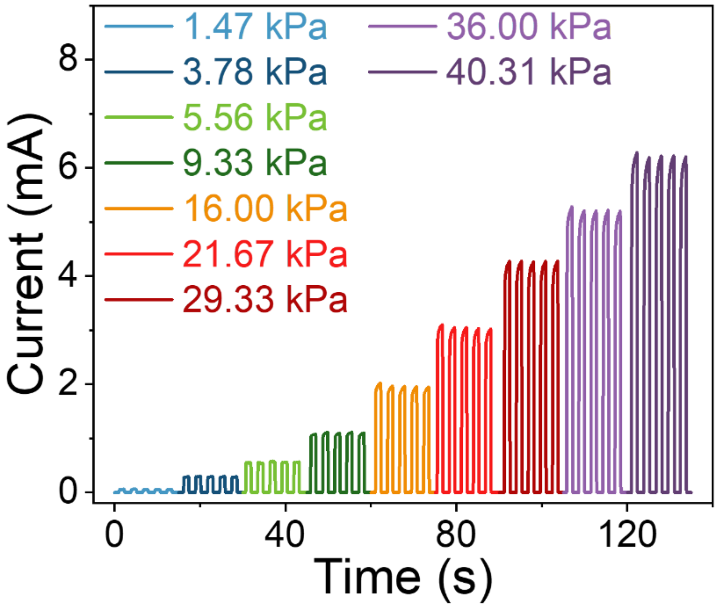

Figure S12. *I*-*t* curves for the MPP piezoresistive sensor at 0.8 mg/mL MXene concentration below 40.31 kPa.

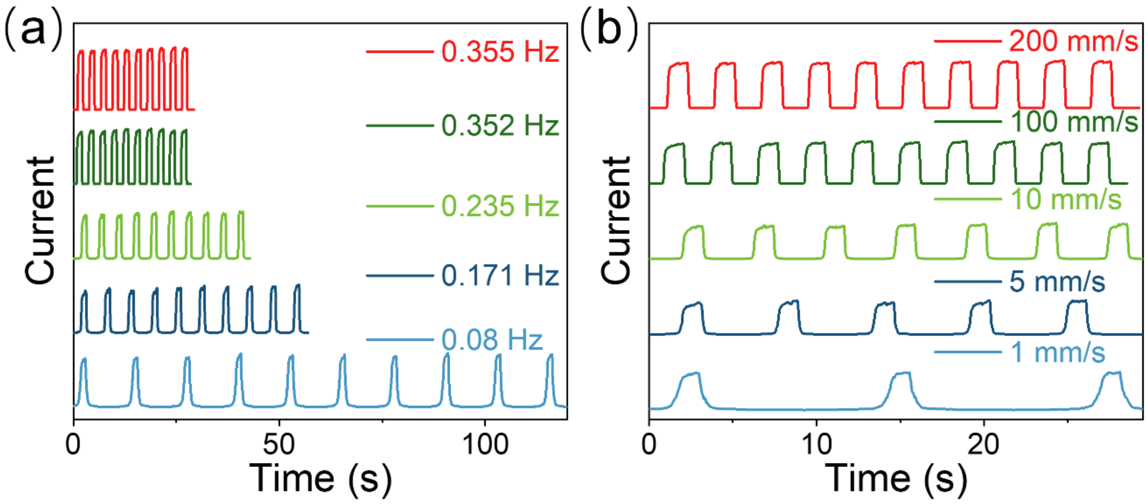

Figure S13. The response of the MPP piezoresistive sensor under the same pressure at different compression (a) frequency and (b) speed.

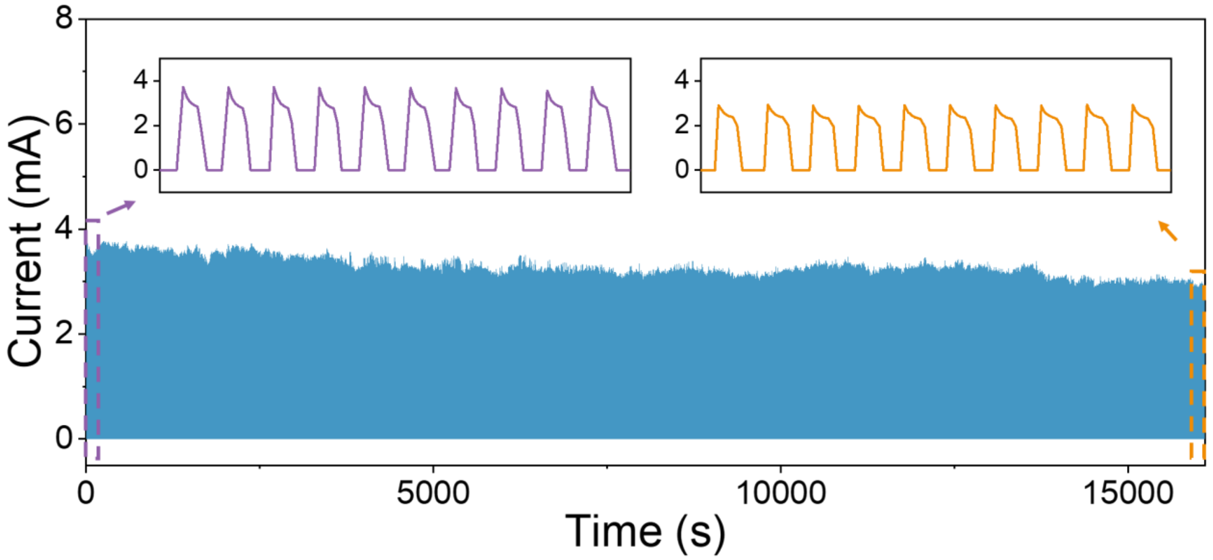

Figure S14. The stability of the MPP sensor at 25.82 kPa during 6000 loading-unloading cycles.

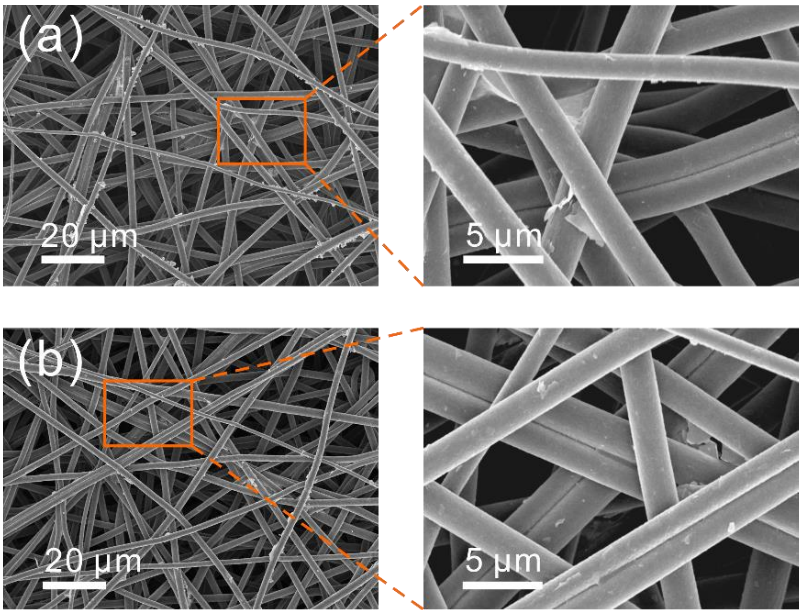

Figure S15. SEM images of the MPP piezoresistive sensor (a) initial state and (b) after 10000 press-release cycles with increasing magnifications.

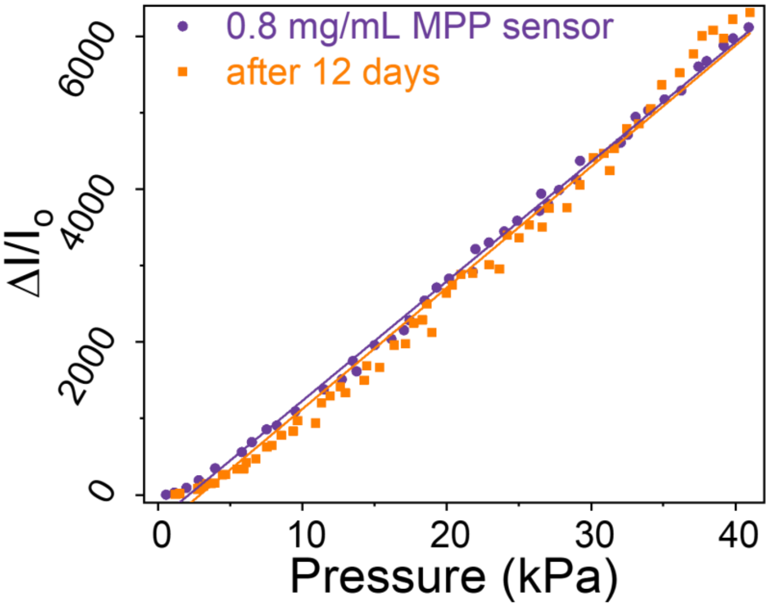

Figure S16. The relative current change ($\Delta I/I_o$) with respect to the pressure for the MPP sensors before and after 12 days.

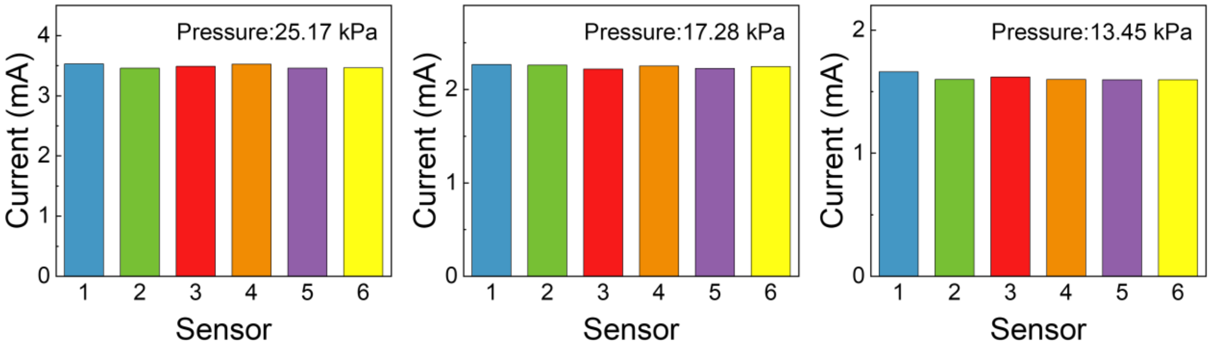

Figure S17. Current response of six MPP sensors under different pressures.

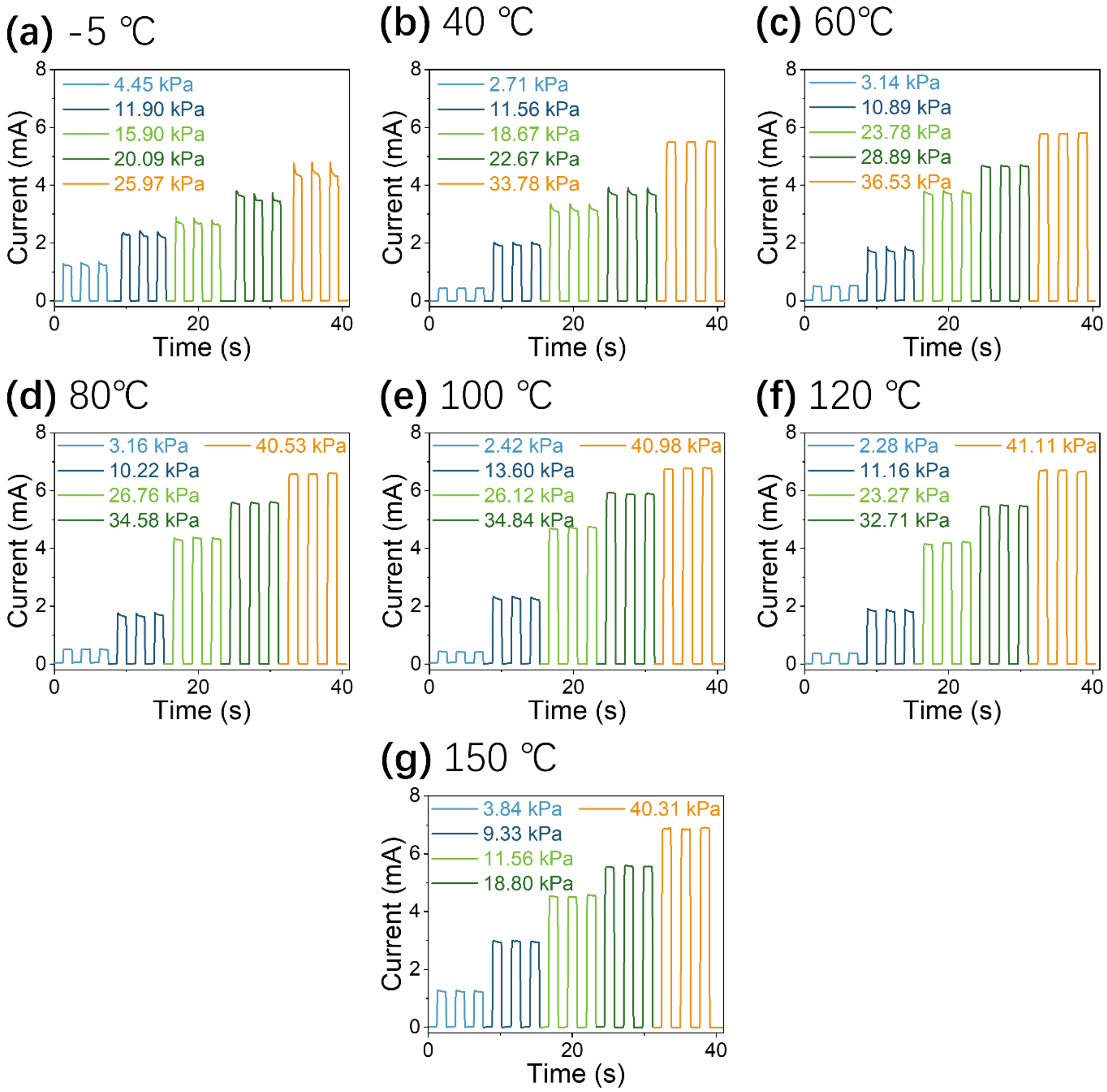

Figure S18. *I-t* curves of MPP piezoresistive sensor at different temperatures from -5 to 150 ℃.

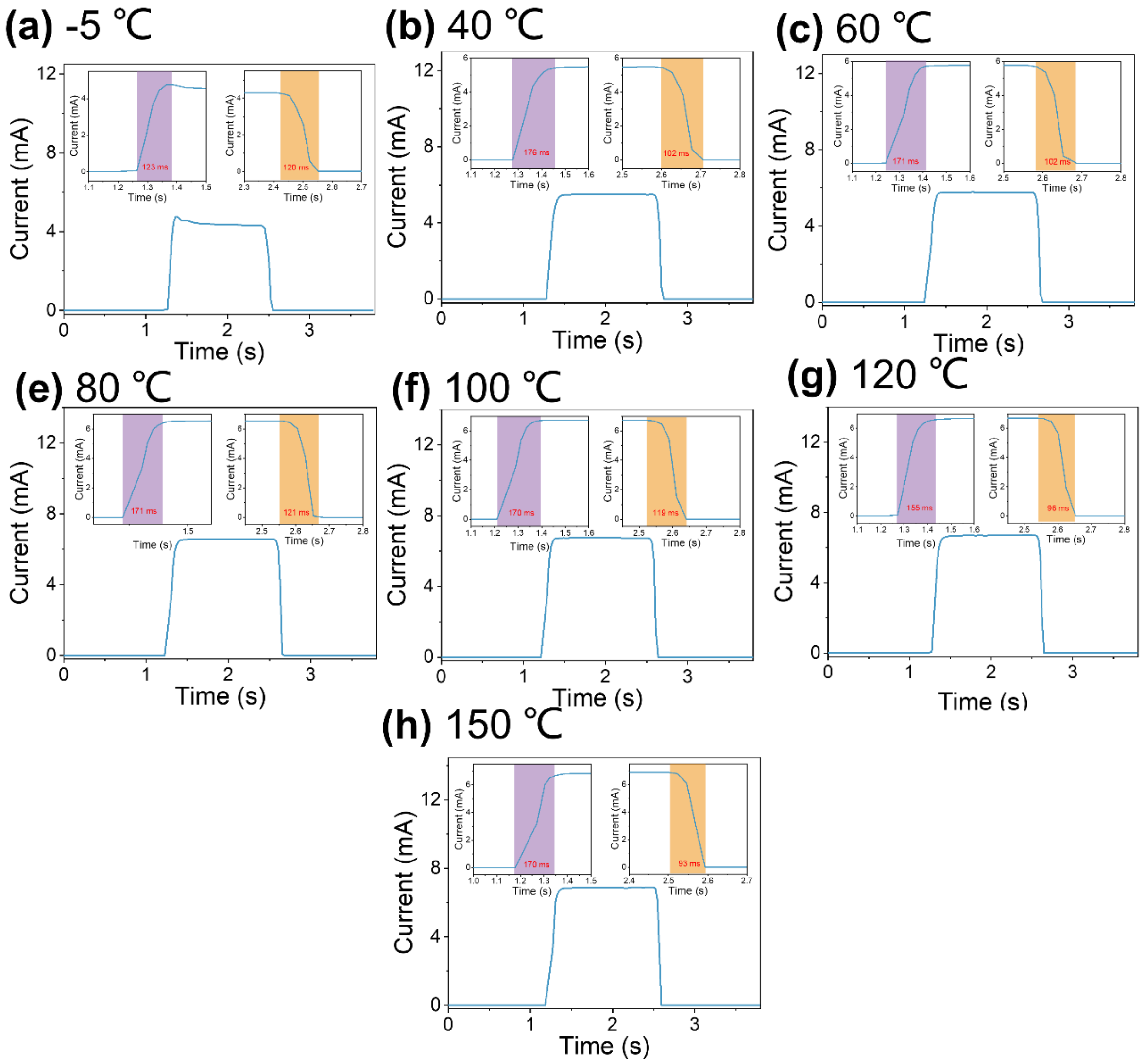

Figure S19. The response time and recovery time of MPP sensor at compression speed of 200 mm/s at different temperature.

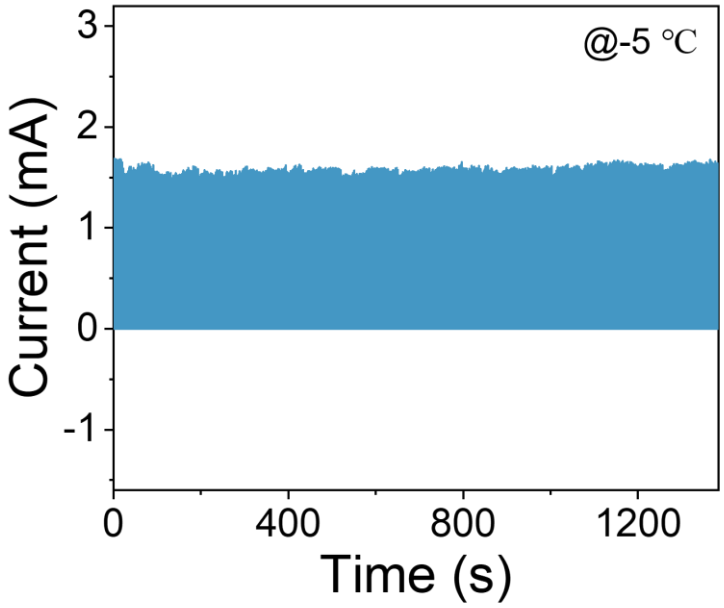

Figure S20. The cyclic performance of the MPP sensor at -5 ℃ over 500 cycles.

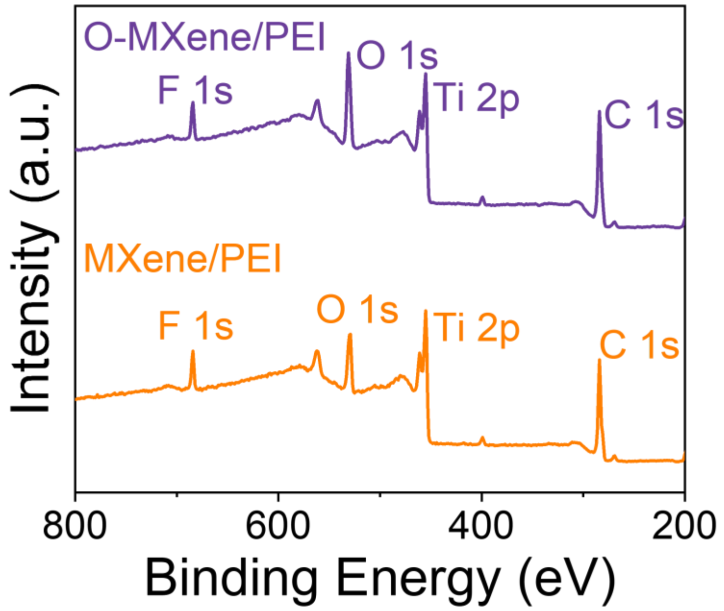

Figure S21. XPS survey spectra of MXene/PEI fibrous network before and after high-temperature tests.

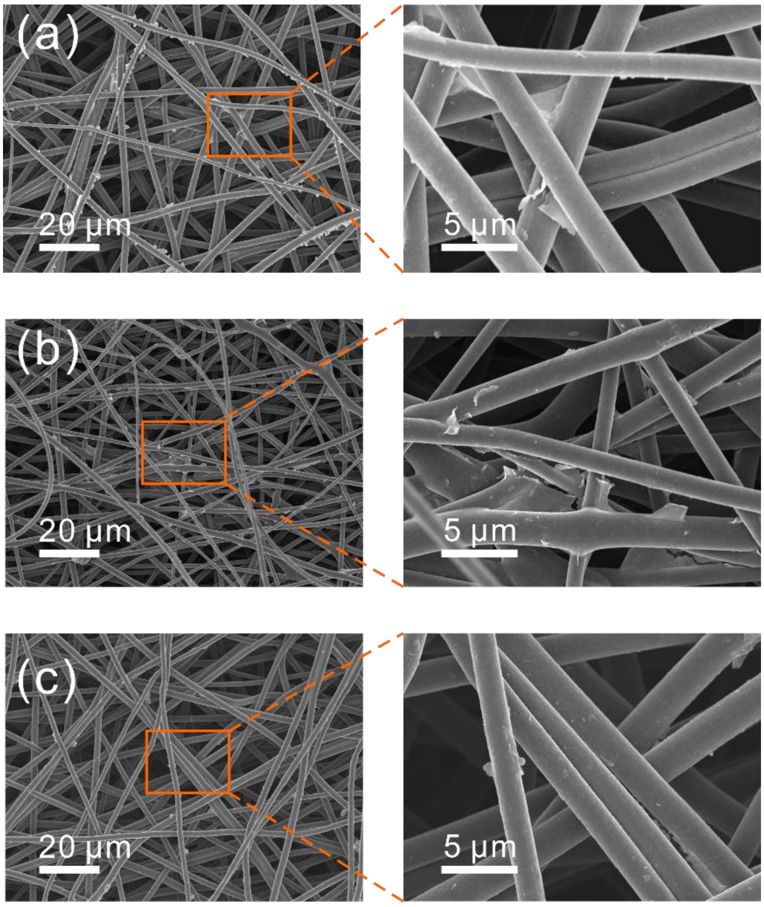

Figure S22. SEM images of the MXene/PEI fibrous network (a) before the high-temperature test, (b) after 60 °C cycling test and (c) after 150 °C test.

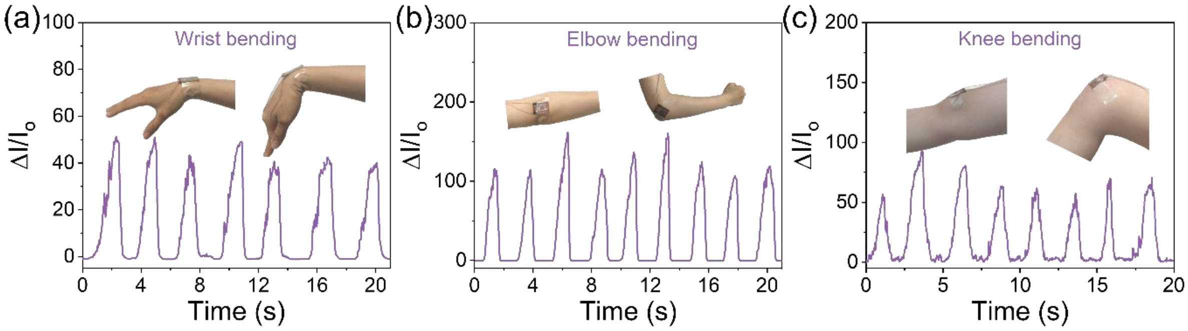

Figure S23. The dynamic response curves of (a) wrist bending, (b) elbow swing, (c) knee bending.

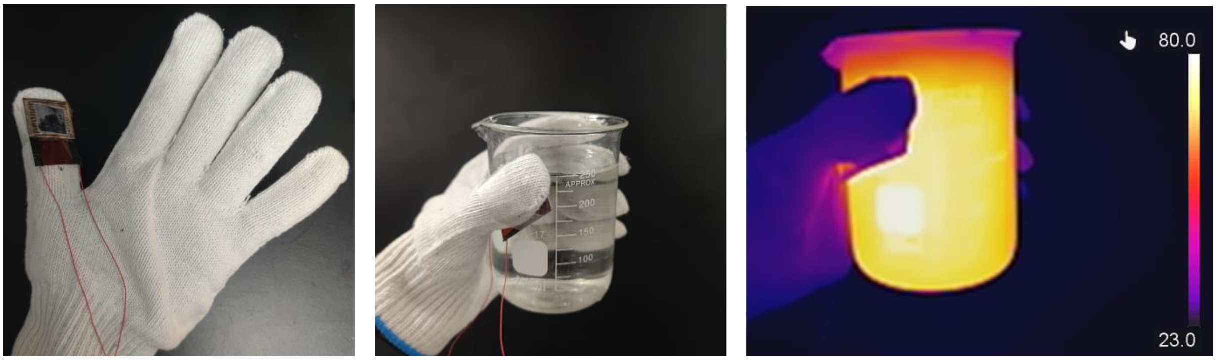

Figure S24. the pictures how the sensors were mounted and how the experiments were tested in **Figure 4g**.

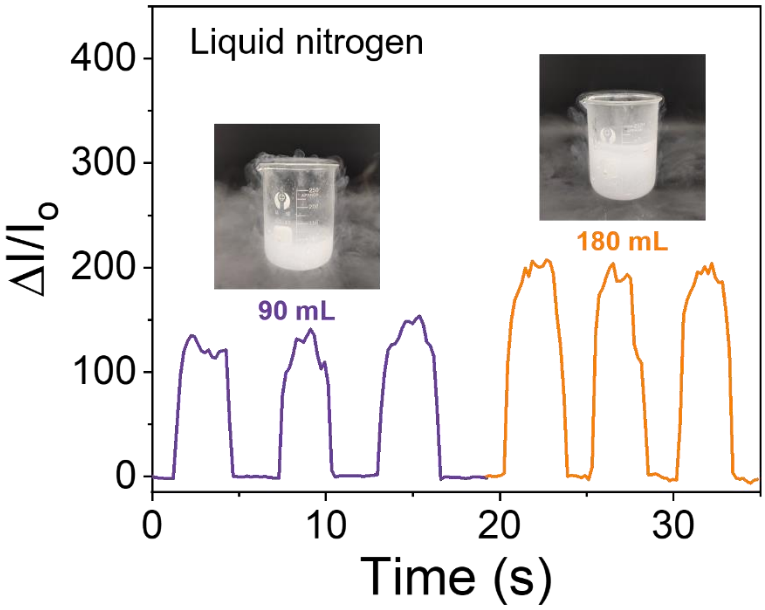

Figure S25. The response curves of glass beaker filled with different volumes of liquid nitrogen.

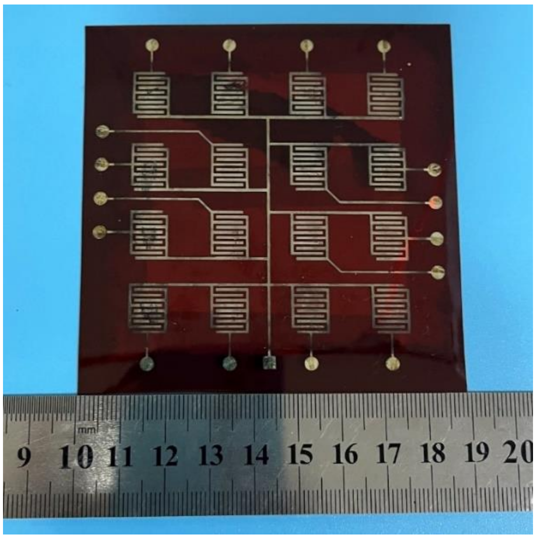

Figure S26. Photograph of the 4×4 array used in **Figure 5**a.

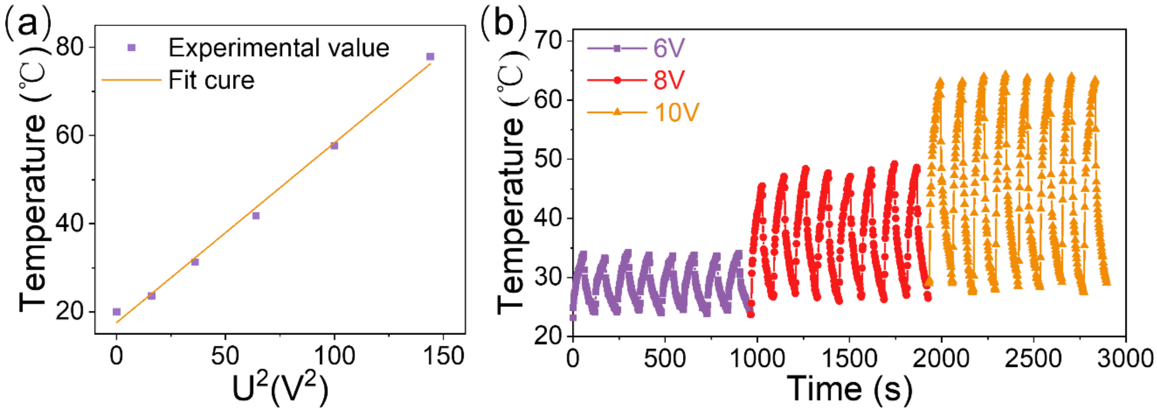

Figure S27. (a) Saturation temperature experimental data and linear fitting of U^2, (b) Heating cycles at applied DC voltages of 6, 8, and 10 V.

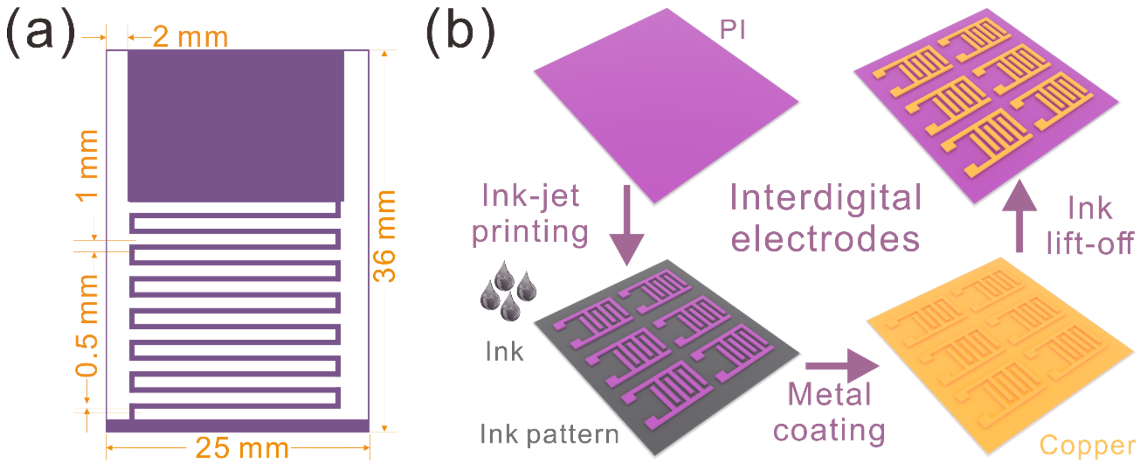

Figure S28. (a) Size parameters and (b) fabrication process of the interdigital electrodes.

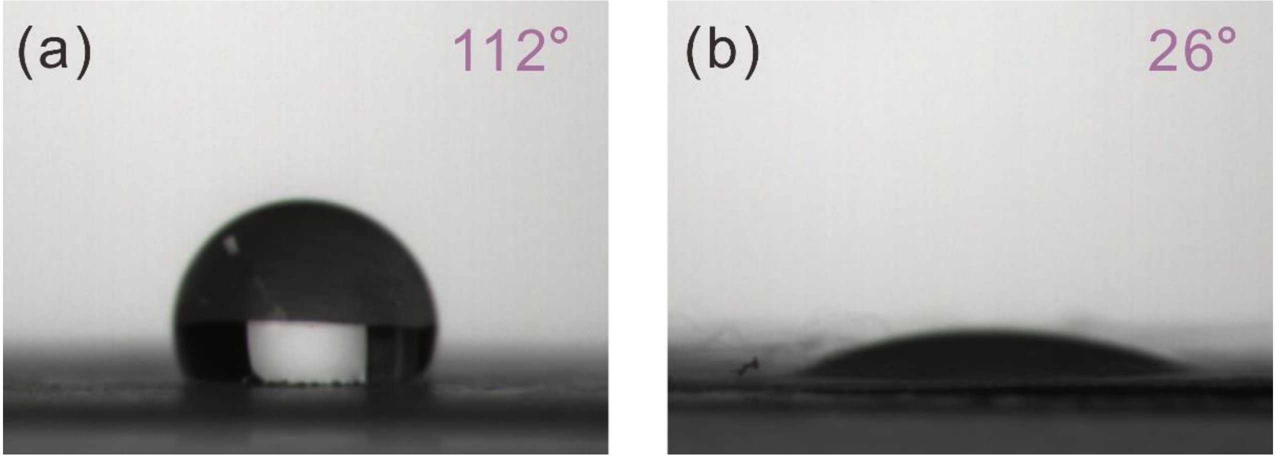

Figure S29. Contact angle measurement. Optical image of water on the surface of PEI fibrous network (a) before and (b) after hydrophilic treatment.

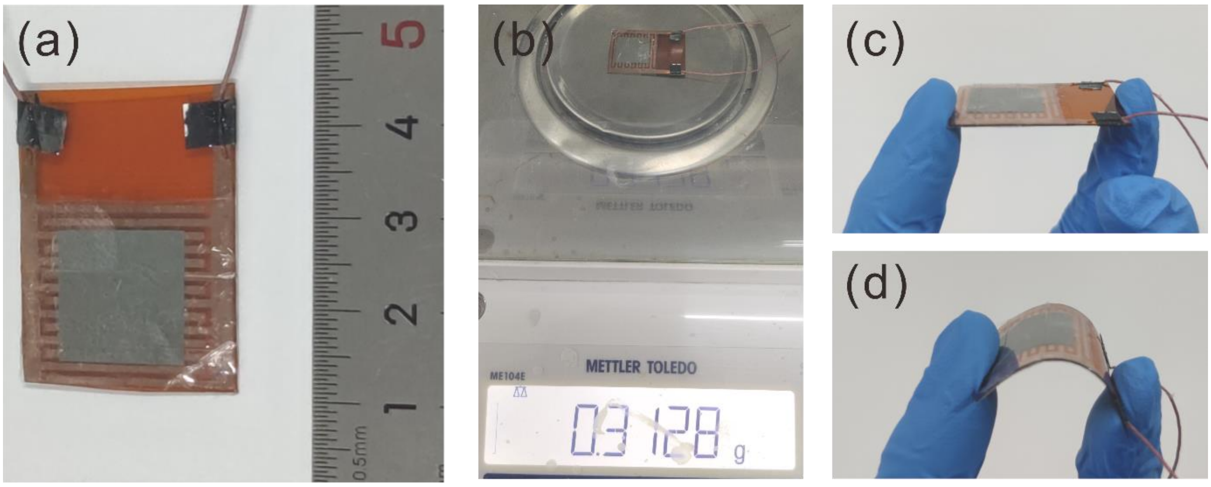

Figure S30. Macroscopic properties of the prepared sensors. (a) Photograph of the assembled MPP sensor with description of its size. (b) Weight (0.3128 g) of the prepared MPP sensor. (c) Side view of the prepared MPP sensor. (d) Flexibility of the prepared MPP sensor.

Table S1. Sensing performance comparison of related piezoresistive sensors.

Ref.	Sensitivity at RT	Stability at RT (cycles)	Electrical signal		Sensitivity at high temperature	Stability at high temperature (cycles)
			Cold	Hot		
[1]	77.61 kPa^{-1} for 0-25 kPa	10000	×	×	×	×
[2]	151.4 kPa^{-1} for 0-4.7 kPa;<br>33.8 kPa^{-1} for 4.7-15 kPa	10000	×	×	×	×
[3]	104 kPa^{-1} for 0.2-7.7 kPa	10000	×	×	×	×
[4]	7.65 kPa^{-1} for 0-3.3 kPa;<br>0.98 kPa^{-1} for 3.3-12.2 kPa;<br>0.24 kPa^{-1} for 12.2-50 kPa	1000	×	×	×	×
[5]	164.75 kPa^{-1} for 0-35 kPa;<br>40.17 kPa^{-1} for 35-100 kPa	7000	×	×	×	×
[6]	134 kPa^{-1} for 0-1.5 kPa;<br>3.5 kPa^{-1} for 1.5-15 kPa；<br>0.7 kPa^{-1} for 15-75 kPa;	8000	×	×	×	×

[7]	0.021 kPa^{-1} for 0-1 kPa;<br>0.054 kPa^{-1} for 1-7 kPa;<br>0.019 kPa^{-1} for 7-17 kPa	200	√	√	×	20 (120 °C)
[8]	×	1000	×	√	×	1000 (250 °C)
[9]	0.00563 kPa^{-1} for 0-3.5 MPa	4000	×	√	0.03866 kPa^{-1} for 0-36 kPa, 0.00285 kPa^{-1} for 30 kPa-6.88MPa at 200 °C	×
[10]	0.14 kPa^{-1} for 0-5 kPa;<br>0.002 kPa^{-1} for 40-85 kPa	1000	√	√	×	×
**Our work**	**156 kPa^{-1} for 40 kPa**	**10000**	**√**	**√**	**80 kPa^{-1} at -5 °C**<br>**156±5 kPa^{-1} from RT to 80 °C;**<br>**104 kPa^{-1} at 100 °C;**<br>**71 kPa^{-1} at 120 °C;**<br>**20 kPa^{-1} at 150 °C**	**2000 (100 °C)**

Reference

[1] Zhang, Y.; Wang, L.; Zhao, L.; Wang, K.; Zheng, Y.; Yuan, Z.; Wang, D.; Fu, X.; Shen, G.; Han, W., *Adv. Mater.*, **2021**, *33*, 2007890.

[2] Cheng, Y.; Ma, Y.; Li, L.; Zhu, M.; Yue, Y.; Liu, W.; Wang, L.; Jia, S.; Li, C.; Qi, T.; Wang, J.; Gao, Y., *ACS Nano*, **2020**, *14*, 2145-2155.

[3] Fu, X.; Wang, L.; Zhao, L.; Yuan, Z.; Zhang, Y.; Wang, D.; Wang, D.; Li, J.; Li, D.; Shulga, V.; Shen, G.; Han, W., *Adv. Funct. Mater.*, **2021**, *31*, 2010533.

[4] Yao, D.-J.; Tang, Z.; Zhang, L.; Liu, Z.-G.; Sun, Q.-J.; Hu, S.-C.; Liu, Q.-X.; Tang, X.-G.; Ouyang, J., *J. Mater. Chem. C*, **2021**, *9*, 12642-12649.

[5] Zhao, L.; Wang, L.; Zheng, Y.; Zhao, S.; Wei, W.; Zhang, D.; Fu, X.; Jiang, K.; Shen, G.; Han, W., *Nano Energy*, **2021**, *84*, 105921.

[6] Li, X.; Fan, Y. J.; Li, H. Y.; Cao, J. W.; Xiao, Y. C.; Wang, Y.; Liang, F.; Wang, H. L.; Jiang, Y.; Wang, Z. L.; Zhu, G., *ACS Nano*, **2020**, *14*, 9605-9612.

[7] Zhao, X.; Wang, W.; Wang, Z.; Wang, J.; Huang, T.; Dong, J.; Zhang, Q., *Chem. Eng. J.*, **2020**, *395*, 125115.

[8] Zhou, H.; Guo, K.; Ma, S.; Wang, C.; Fan, X.; Jia, T.; Zhang, Z.; Xu, H.; Xing, H.; Wang, D.; Liu, C., *Chem. Eng. J.*, **2022**, *432*, 134431.

[9] Jia, M.; Yi, C.; Han, Y.; Wang, L.; Li, X.; Xu, G.; He, K.; Li, N.; Hou, Y.; Wang, Z.; Zhu, Y.; Zhang, Y.; Hu, M.; Sun, R.; Tong, P.; Yang, J.; Hu, Y.; Wang, Z.; Li, W.; Li, W.; Wei, L.; Yang, C.; Chen, M., *Adv. Sci.*, **2022**, *9*, 2105738.

[10] Liu, H.; Chen, X.; Zheng, Y.; Zhang, D.; Zhao, Y.; Wang, C.; Pan, C.; Liu, C.; Shen, C., *Adv. Funct. Mater.*, **2021**, *31*, 2008006.
